# Longitudinal changes in hand hygiene adherence among healthcare workers during the COVID-19 pandemic, Dominican Republic

**DOI:** 10.1371/journal.pwat.0000231

**Published:** 2024-05-10

**Authors:** C. Daniel Schnorr, Kathryn W. Roberts, Eulogia C. Payano, Paloma Martínez Guzmán, Michael de St. Aubin, Matthew Lozier, Salome Garnier, Devan Dumas, Kelsey McDavid, Cecilia Jocelyn Then Paulino, Ronald Skewes-Ramm, Christina Craig, Emily Zielinski Gutierrez, William Duke, Eric Nilles

**Affiliations:** 1 Department of Emergency Medicine, Brigham and Women’s Hospital, Harvard University, Boston, Massachusetts, United States of America; 2 Infectious Disease and Epidemic Program, Harvard Humanitarian Initiative, Cambridge, Massachusetts, United States of America; 3 Department of Emergency Medicine, Harvard Medical School, Boston, Massachusetts, Unites States of America; 4 Ministry of Health and Social Assistance, Santo Domingo, Dominican Republic; 5 Division of Food, Waterborne, and Environmental Diseases, Centers for Disease Control and Prevention, Atlanta, Georgia, United States of America; 6 Dirección General de Epidemiología, Santo Domingo, Dominican Republic; 7 Central America Office, Centers for Disease Control and Prevention, Guatemala City, Guatemala; 8 Universidad Nacional Pedro Henríquez Ureña, Santo Domingo, Dominican Republic

## Abstract

Hand hygiene (HH) can reduce transmission of healthcare-associated infections (HAIs) in healthcare facilities and is especially important in low- and middle-income countries where HH infrastructure may be insufficient and the burden of HAIs is highest. At baseline, we assessed HH infrastructure and practices among healthcare workers (HCWs) at two large hospitals in the Dominican Republic during the COVID-19 pandemic. HCWs were observed for HH adherence (HHA) (defined as the use of alcohol-based hand rub (ABHR) or hand-washing with soap and water) before and after patient contact and donning new gloves before patient contact. The baseline assessment was repeated following implementation of local production and distribution of ABHR and a HH promotion campaign. Descriptive analyses and regression models evaluated predictors of HHA and glove use. Cumulative HHA was 18.9%. While patient-care areas with a functional HH resource increased from 47% at baseline to 92% after the intervention, HHA declined from 23.0% to 16.7%. HHA was higher after patient contact (aOR = 5.88; 95% CI = 4.17–8.33), during a period of increased COVID-19 risk (aOR = 1.69; 95% CI = 1.05–2.77), during invasive patient contacts (aOR = 1.64; 95% CI = 1.23–2.17) and when gloves were not used (aOR = 1.25; 95% CI = 1.01– 1.56). The negative association between glove use and HHA diminished when access to HH resources increased. New gloves were donned before 39.6% of patient contacts. Glove use was higher among nurses (aOR = 7.12; 95% CI = 3.02–16.79) and during invasive contacts (aOR = 4.76; 95% CI = 2.27–10.0). While access to HH resources increased after the interventions, HHA did not increase. HHA was lower when COVID-19 risk was lower. Findings from this study may guide future efforts to increase HHA among HCWs.

## Introduction

Healthcare-associated infections (HAIs) are the most common complications affecting hospitalized patients, leading to increased antimicrobial resistance, healthcare costs, disability, and death [1]. Effective hand hygiene (HH) in health care facilities (HCFs) via handwashing with soap or using alcohol-based hand rub (ABHR) with at least 60% alcohol content is considered the primary measure for preventing HAIs and the spread of antimicrobial resistance [2–4].

Though increased adherence with effective HH protocols is strongly linked to a reduction in HAIs [3, 5], achieving and maintaining high levels of HH adherence among healthcare workers (HCWs) remains a considerable challenge to healthcare systems worldwide [6, 7]. Adherence to HH protocols among HCWs increased during the COVID-19 pandemic, though may have fallen subsequently as the perceived threat subsided, consistent with reports from past outbreaks with respiratory pathogens [8–11].

The burden of HAIs is higher in low- and middle-income countries (LMICs) [12]. Inadequate water, sanitation, and hygiene (WASH) infrastructure likely contributes to this disparity. A recent assessment in 78 LMICs estimated that 50% of HCFs lacked access to an improved water source and 39% had inadequate handwashing facilities [13]. In recognition of these challenges, the World Health Organization (WHO)/United Nations Children’s Fund (UNICEF) Joint Monitoring Program (JMP) included 100% coverage of WASH infrastructure in HCFs in LMICs by 2030 among its Sustainable Development Goals [14]. Local production of ABHR has gained attention as a cost-effective means for addressing gaps in HH facilities in LMICs [15], and the WHO has published guidelines to facilitate implementation [16]. While increased access to functional HH resources has been linked to improved HH practices among HCWs, the best results are seen when such improvements are paired with other HH promotion activities at the institutional level in a multimodal approach [5, 6].

The Dominican Republic (DR) is an upper-middle-income country that faces challenges in improving WASH in its HCFs. Limited published data suggest risks of post-surgical infections in DR are high [17]. Data concerning HH behaviors among HCWs in DR are limited. The COVID-19 pandemic exacted a heavy toll in cases and deaths and strained the healthcare system in DR [18]. To better understand HH behaviors among HCWs in the pandemic context and to inform mitigation and response measures, we evaluated HH practices and associated factors at two HCFs in DR during the COVID-19 pandemic. A baseline evaluation of HH infrastructure and behaviors informed interventions to improve HH practices and access to ABHR where gaps were identified. Follow-up assessments evaluated changes in HH behaviour after these interventions and during different stages of the COVID-19 pandemic. Findings will be used to inform future directions for infection prevention and control in healthcare settings.

## Methods

### Hospital selection and study design

The two participating hospitals were selected based on their engagement with the Centers for Disease Control and Prevention (CDC) Central America Regional Office in acute febrile illness surveillance. Hospital 1 is a 93-bed hospital located in the provincial capital of a mountainous, agricultural area which employed 470 clinical staff and performed approximately 3,000 outpatient consultations and 400 admissions monthly at the beginning of the study period (April, 2021). Hospital 2 is a 250-bed regional hospital serving multiple coastal provinces, which employed 513 clinical staff and averaged over 9,000 outpatient consultations and 800 admissions monthly at the initiation of the study period (May, 2021). Both hospitals included operating rooms, intensive care units, labor and delivery units, adult and pediatric wards, as well as specialized COVID-19 inpatient units. Both hospitals have access to an improved water source via piped water supplied by municipal governments, though staff at each site reported regular disruptions in service.

**Fig 1.**
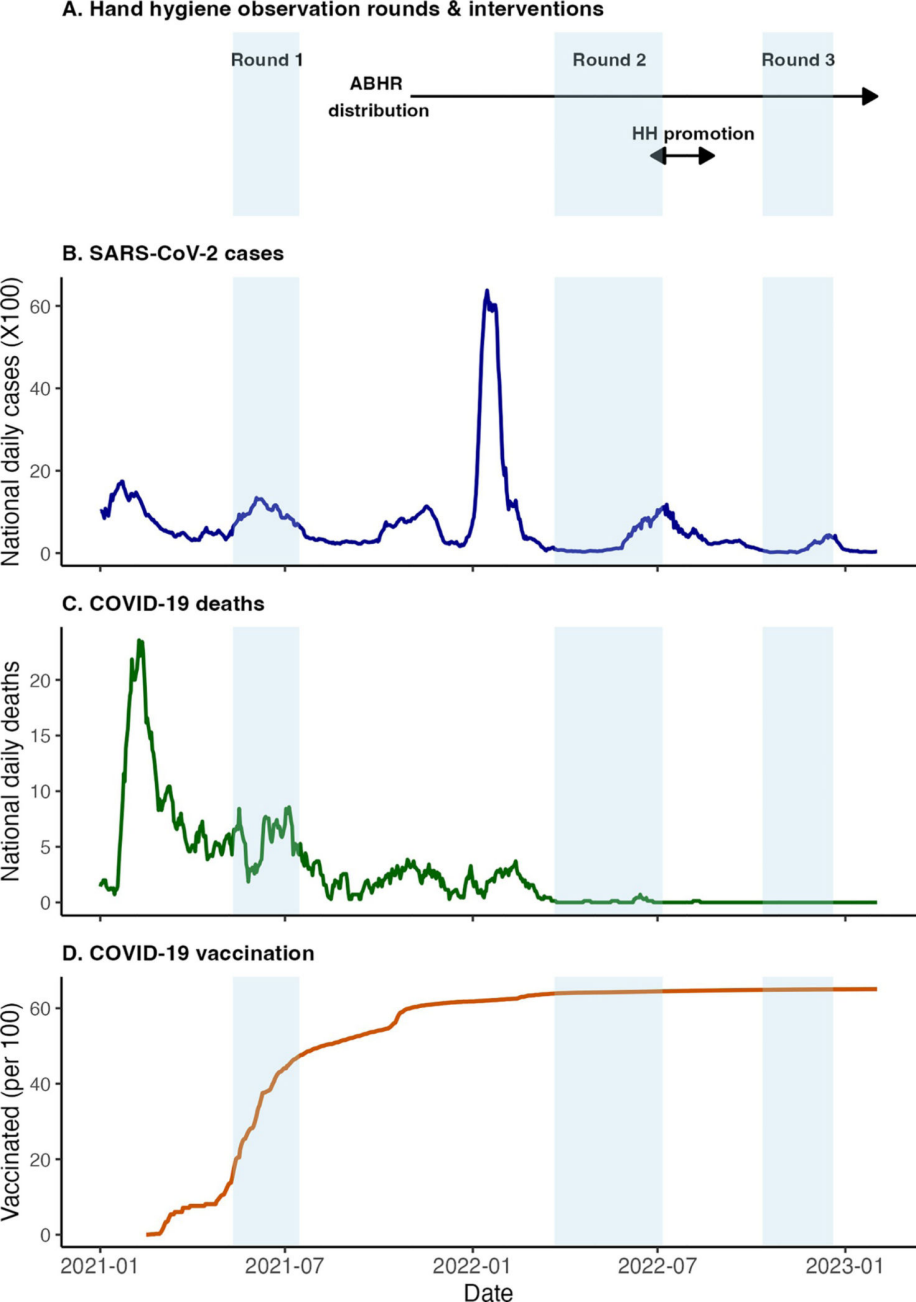
Study timeline in relation to key COVID-19 indicators [18].

The baseline evaluation to assess HH infrastructure and HH behavior at participating hospitals consisted of a HH resource assessment at points of care, round 1 of HH observations, and qualitative interviews with hospital staff and took place during May to September 2021. Ten semi-structured interviews were conducted with hospital staff from each hospital in September 2021. While pertinent findings from these interviews are cited in the results and discussion section, a full report on that component of the evaluation is published elsewhere [19]. Data from the baseline HH resource assessment and HH observations were pooled with data from other LMICs and also previously published [20, 21]. The baseline evaluation informed the development of two interventions aimed at improving HH practices among HCWs: local production and distribution of ABHR and a HH promotion campaign. The HH resource assessment was repeated at the end of the study (January–March, 2023) to assess changes in HH resources at points of care. HH observations were repeated after the ABHR intervention (round 2: March–July, 2022) and again after the HH promotion campaign (round 3: October–December, 2022) to assess changes in HH behaviors following the interventions and at different stages during the COVID-19 pandemic (Fig 1).

### Hand hygiene resource assessment

To understand gaps in HH infrastructure, we assessed the presence and functionality of hand hygiene resources (HHRs) in all patient-care areas (PCAs) at baseline and after an ABHR production and distribution intervention using a standardized electronic data collection form. The baseline assessment was conducted from 4/6/2021–9/23/2021 and the follow-up was conducted from 1/11/2023–3/16/2023. These assessments were performed by direct observation of PCAs. For a small number of PCAs that were not accessible at the time of assessment, enumerators collected information from hospital staff with knowledge of the areas. HHRs were defined as either a hand-washing station or ABHR dispenser. To be considered functional, handwashing stations needed a functional tap, running water, and soap available within six feet, and ABHR dispensers needed a functional dispenser mechanism and ABHR present in the device.

### Hand hygiene adherence observations

We conducted cross-sectional HH observations at baseline and during two follow-up periods to measure the rate of Hand Hygiene Adherence (HHA) among HCWs. HHA was defined as either handwashing with soap and water or ABHR use. The target sample size was 65 HCWs at each facility per observation round to provide power to detect a 10% change in overall HHA. The eligible HCW population was segmented by department then sampling proportional to size was conducted according to the number of staff in each department at baseline. Representation from each department was kept constant between rounds. Random sampling was repeated for each round. Successive rounds may have contained repeat respondents, though this was not tracked. To minimize observation bias, HCWs were informed that we were assessing healthcare practices and patient-provider interactions but did not specify that we were investigating HH practices. HCWs who provided verbal consent to participate were observed during five consecutive patient contacts or until one hour had elapsed, whichever was shorter.

Observers were trained remotely in their preferred language (Spanish in all cases) using WHO materials. Training included a lecture component, small-group question-and-answer sessions and practical role play to identify strategies and potential challenges. During routine patient care activities, based on WHO’s five moments of hand hygiene, observers recorded potential HH opportunities before and after patient contact. Observers recorded each instance of HH along with the method as well as each missed opportunity for HH. They also recorded the donning of clean gloves prior to patient contact. Observers did not record whether or not gloves were indicated for a given patient contact, nor did they record whether HH was performed before or after donning or doffing of gloves for patient contacts in which both gloves and HH were used. To assess for factors associated with HHA, observers recorded the profession of the HCW being observed, whether each HH opportunity occurred before or after patient contact, and whether the patient contact type was invasive or non-invasive. An invasive contact was defined as any interaction involving contact with broken skin or a medical procedure that enters the body, such as treating a wound, inserting an intravenous catheter, administering a vaccine, or drawing blood.

### Local production and distribution of ABHR

**Fig 2.**
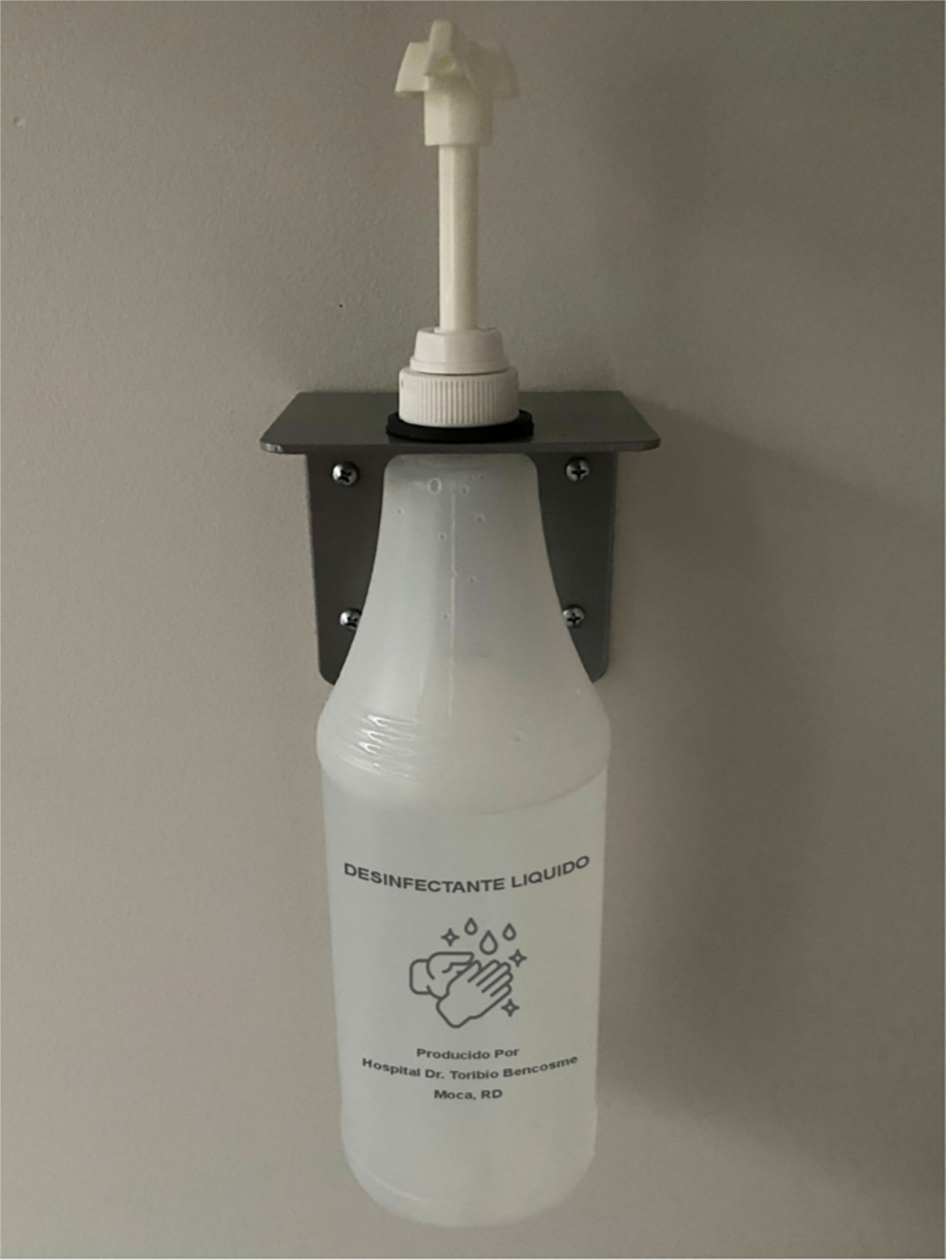
Photograph of dispensers for locally produced ABHR.

Selected hospital staff were trained to produce Isopropyl alcohol-based ABHR on-site in accordance with WHO standards [16]. Staff adhered to WHO safety guidelines and were trained to report all safety events to the principal investigator. Internal and external quality control procedures were employed to ensure 70%−80% concentration of alcohol for all batches. The ABHR product was portioned into one-liter bottles with pump dispensers that were fixed to the walls of PCAs with metal brackets (Fig 2). Distribution points were selected based on information collected during the baseline HHR assessment with the goal of installing at least one ABHR dispenser at each PCA and nursing station. Dispensers were monitored three times per week by direct observation to ensure continued functionality and supply of ABHR from initiation of the intervention prior to round 2 of hand hygiene observations, through the conclusion of the study period at the end of round 3 of observations.

### Hand hygiene promotion campaign

At each hospital, we conducted 10 qualitative in-depth interviews with hospital staff concerning motivators for and barriers to HHA to inform hospital-wide behavior change communication campaigns aimed at increasing HHA. Staff education specifically emphasized that ABHR use and hand washing with soap and water are effective HH methods and that HH should be performed prior to donning gloves and after doffing. Visual materials were message tested in each facility and then selected materials were posted throughout each facility beside the ABHR dispensers installed in the first stage of the intervention (July, 2022). Hand hygiene champions were purposively selected from various departments, trained in HH promotion, and supported by study staff (June–August, 2022). The champions promoted specific hand hygiene messages each week for six weeks, based on knowledge and practice gaps identified in baseline interviews.

### Ethical considerations

The study protocol and informed consent forms were approved by Institutional Review Boards and delegated authorities of Mass General Brigham, Universidad Nacional Pedro Henriquez Ureña, Hospital 1, Hospital 2 and Consejo Nacional de Bioética en Salud de la República Dominicana. All study subjects provided verbal informed consent after listening to detailed study information read by study staff from a standardized form. The recruitment period for human subjects began on 4/6/21 and concluded on 3/15/23.

### Data analysis

Differences in proportions of PCAs containing HHRs from baseline to follow-up were assessed using Pearson’s chi-squared test. Composite HHA was reported as the proportion of total hand hygiene opportunities (before and after patient contact) in which either ABHR or hand washing with soap were used. To assess for predictors of HHA and glove use while accounting for the observer, we fit a Generalized Estimating Equations (GEE) model using the observer as the clustering variable. An autoregressive correlation structure was selected in the GEE model for yielding the lowest quasi-likelihood under the independence model criterion (QIC). Analyses were performed using the R statistical programming language (R version 4.1.3, 2022–03-10). The outcome variables examined were HHA and glove use. We included glove use as an outcome variable because we were interested in the relationship between glove use and HHA, which may in turn inform hand hygiene education interventions. Predictive variables included in all analyses were observation round, contact-type (invasive or non-invasive), Hospital, and HCW profession. Additionally, glove use and the relationship of HH opportunity to patient contact (before or after) were included as predictive variables in the analysis of predictors of HHA. In order to compare our findings with similar studies in which glove use was not included, we also analyzed predictors of HH while excluding the effect of glove use. To better understand the effects of the two interventions, each of these analyses were run using Round 1 as the reference, using Round 2 as the reference, and with Round 1 excluded.

## Results

### Hand hygiene resource assessment

At baseline 79 (47%) PCAs contained at least one functional HHR (64 (38%) with functioning hand washing stations, 43 (25%) with functioning ABHR dispensers, and 27 (16%) with both). Handwashing stations were the most common HHR but less than half were functional. The most common reasons for a handwashing station to be categorized as non-functional were lack of soap (N = 78, 53%), lack of running water (N = 37, 25%), and non-functioning tap (N = 31, 21%). At baseline, 52 (31%) PCAs contained an ABHR dispenser, 43 (83%) of which were functional. At the follow-up assessment, 173 (92%) PCAs contained a functional HHR. There were increases in the percentage of PCAs containing any functioning HHR, any ABHR dispenser, and a functioning ABHR dispenser (Table 1). No safety events were reported pertaining to ABHR production and distribution.

### Hand hygiene observations

We observed a total of 408 HCWs during 2,029 patient contacts. Of these, complete before- and after-patient contact data were available for 2,011 and 2,025 contacts, respectively, for a total of 4,036 HH opportunities that were used for analyses. The number of observations were similar between the two hospitals (Hospital 1 N = 1,976; Hospital 2 N = 2,060), and across each of the three observation periods (Round 1 N = 1,354, Round 2 N = 1,366, Round 3 N = 1,316) (Fi 1). The three observation periods corresponded to substantially different levels of COVID-19 risk as indicated by nationally reported SARS-CoV-2 cases, deaths, and vaccination coverage (Fi 1), with the highest risk during the baseline evaluation (Round 1) and lowest during the final evaluation (Round 3).

Composite HHA across all timepoints was 18.9%. HHA before and after patient contact was 7.2% and 30.3%, respectively (Table 2). Before patient contact, HCWs were more likely to use ABHR than handwashing with soap in all observation periods. After patient-contact, HCWs were more likely to use ABHR than handwashing with soap in Round 3 only. New gloves were donned before 39.6% of patient contacts. During patient contacts in which gloves were used, staff practiced HH during 0.8% of HH opportunities before patient-contact and 14.2% of HH opportunities after patient-contact (Table 2).

### Predictors of hand hygiene adherence

Multivariate analyses found that HHA was lower during Round 3 (17.2%) than Round 1 (23.0%) and trended downward in Round 2 (16.3%) vs Round 1. HHA was lower before patient contact, during non-invasive procedures, when gloves were used, and at Hospital 1 (Table 3). When we removed glove use from our analysis of predictors of HHA, doctors were more likely than nurses to practice HH (S1 Appendix). When Round 2 was used as the reference, HHA in Round 3 was similar (aOR 0.94 (0.59–1.52), p = 0.807). When Round 1 was excluded from the analysis, the negative effect of glove use on HHA was no longer statistically significant (aOR 0.90 (0.67–1.22), p = 0.517) while all other predictors of HHA remained unchanged.

### Predictors of glove use

In multivariate analysis, new glove use was higher during invasive contacts, at Hospital 2 and among nurses as compared with doctors (Table 4). Glove use was lower during Round 2 than Round 1. When Round 2 was used as the reference, glove use trended higher in Round 3 (aOR 1.30 (0.83–2.04) p = 0.260). When Round 1 was excluded from the analysis, glove use was higher in Round 3 versus Round 2 (aOR 1.82 (1.02–3.23), p = 0.043) and predictors of glove use remained unchanged.

### Semi-structured interviews

Notable findings include a high level of knowledge of HH methods and acceptability for both ABHR and washing hands with soap and water, with a general preference for soap and water. Interviews identified key drivers of HH practice, which included staff’s desire to protect themselves and their families and to a lesser extent to protect their patients. Barriers to HH practice primarily included lack of time and inconsistent access to HH supplies. In situations when time was limited, staff described skipping HH or using gloves as an alternative to HH to save time. A full report on these interviews is published elsewhere [19].

## Discussion

This study examined HH resources, HHA, and glove use among HCWs at two large hospitals in DR at various timepoints during the COVID-19 pandemic. The percentage of PCAs containing a functional HHR increased from 47% to 92% following the local production and distribution of ABHR. Despite this increase in HHR and a HH promotion campaign, HHA decreased over the course of the study from the baseline of 23%, to 16.3% in Round 2 and 17.2% in Round 3. HHA was higher in Round 1, after patient contact, during invasive procedures, and when gloves were not used. In addition to examining HHA, we also explored predictors of glove use. New gloves were donned before 39.6% of patient encounters.

Our study had several limitations. Due to the convenience sampling method and a small number of study sites, our findings cannot be generalized to other facilities of similar size in the DR. A second limitation is that we did not record the availability of HHR’s or gloves at the moment of each HH opportunity. A substantial difference in glove use between the two hospitals may be at least partially attributable to the unequal availability of fitting gloves. While it was not directly measured, staff from Hospital 2 reported stocking gloves of all sizes in most PCA’s, while staff from Hospital 1 reported providing only one glove size and stocking them at central locations, such as nursing stations. Lastly, efforts to achieve 100% coverage of PCAs with a functional HHR were impeded by a full renovation of Hospital 2 between baseline and follow-up.

While the results may not be generalizable to other countries or other settings in DR, the low rate of HHA observed among HCWs is similar to that seen in other LMICs during the same period (range 23%–52% in one study [21]). Increased HHA after patient contact and during invasive contacts is a commonly reported observation [6, 21–24]. Though the majority of similar studies conducted before the COVID-19 pandemic report greater HHA among nurses compared with doctors [6, 7, 22], we observed the opposite. In fact, when excluding the role of glove use, doctors were significantly more likely than nurses to practice HH (S1 Appendix). This finding is consistent with a study from five LMICs during the same time period, which included data from our study [21], and may indicate a shift in HH behavior attributable to changing perceptions of COVID-19 risk as the pandemic progressed. While increased workload among nurses during the pandemic may contribute to lower HHA [25], our findings suggest that glove use plays an important role in HH behavior. Compared with doctors, nurses were seven times more likely to don new gloves prior to a patient encounter, and glove use was associated with reduced HHA in this and other studies [3, 26–28]. These findings suggest that glove use is considered by many HCWs as a reasonable alternative to HH. However, glove use alone does not constitute HHA. Gloves can be contaminated by hands in the process of donning and still transmit HAIs [29]. Therefore, hand washing with soap and water or using ABHR is required, regardless of glove use.

In multivariate analysis of three rounds of HH observations, glove use was a negative predictor of HHA. One possible explanation in a setting where access to HH resources at the point of care was not always reliable, is that staff were more likely to use gloves when HH resources were not available. The effect of glove use on reduced HHA was most pronounced during the Round 1 of hand hygiene observations when access to HH resources was lowest. During later rounds when increased HH resources were available, there was no association between glove use and HHA. Additional explanations for decreased HHA seen with glove use include a lack of time and lack of knowledge concerning the potential to contaminate gloves when donned with unclean hands. In qualitative interviews conducted at baseline, HCWs displayed high levels of knowledge concerning effective HH methods, but some acknowledged that “in situations where time is limited, [⋯] they save time by changing gloves without practicing hand hygiene” [19].

Our data suggest that real or perceived risk from infectious disease outbreaks may be a stronger predictor of HHA among HCWs than either increased access to HH resources or a multimodal HH promotion campaign. In qualitative interviews with staff at the study hospitals, the desire to protect themselves and their households was the most frequently cited motivation for practicing HH. The desire to protect patients and coworkers was cited less frequently [19]. In similar studies, self-protection is a frequently cited motivator for HHA [30]. This may explain why HHA was highest when the risk to the HCW was elevated, including early in the pandemic when COVID-19 mortality was high (Fig 1), during invasive contacts, after patient contact, and when gloves are not used. This also helps to explain why glove use was more common than HH even after access to HHRs increased. Gloves provide meaningful if incomplete protection for the HCW compared with minimal protection for the patient. Especially when time is limited, and even when HHRs are available, HCWs may feel the need to choose between gloves and HH before a patient contact. Using both requires additional time to allow hands to fully dry before donning gloves. Staff were unlikely to both don new gloves and practice HH before a patient contact even after access to HHRs increased. This finding presents an opportunity to increase HHA among HCWs before patient contact, either by specifically targeting encounters when gloves are used for HH promotion, or by highlighting patient encounters in which gloves are used unnecessarily.

A preference for handwashing over ABHR among the study population may be a contributing factor to why HH did not increase following our interventions. In qualitative interviews, staff in all roles across both study sites expressed a preference for handwashing over ABHR [19]. Following the ABHR intervention, while the availability of ABHR dispensers at PCAs increased significantly, the percentage of PCAs with a functioning handwashing station remained constant. When COVID-19 risk declined, the fall in HHA was driven almost entirely by decreased handwashing, while ABHR use after patient contact actually increased. Without the ABHR intervention, HHA in follow-up assessments may have decreased further than what was observed. Given staff preferences, the intervention may have been more effective if paired with improvements in handwashing facilities, though this would have been difficult given the cost and dependence on a consistent external water source.

Similarly, though HHA did not increase following the HH promotion campaign, we believe the interventions were beneficial. When we compared only the follow-up observation periods when HHR availability was maintained at high levels (rounds 2 and 3), COVID-19 risks as defined by mortality declined. Yet, despite the decreased risk to HCWs, HHA remained similar between the two rounds. A shift in staff preference for HH method to ABHR following the promotion campaign may have contributed, as well as HCWs preference for glove use. HCWs used gloves without HH before patient contact more frequently than they practiced HH before patient contact in all rounds of observation, but notably, after falling significantly between rounds 1 and 2, glove use increased between rounds 2 and 3. Given that COVID-19 risk continued to fall during the latter period, the increase may be attributable to the campaign. Though the campaign emphasized the importance of ABHR or handwashing even when using gloves, and explicitly stated that glove use is not HH, it also highlighted the persistent risks due to COVID-19 and other HAIs, which could prompt staff to increase self-protective behaviors.

The proportion of patient encounters in which HCWs both practiced HH and used gloves before contact remained low in Round 3. Since HCWs in our study prioritized gloves as a method of personal protection, future HH promotion efforts may promote strategies for adhering to HH protocols even when gloves are used. Alert fatigue, which may have increased with increased public health messaging attributable to the COVID-19 pandemic, may also partially explain why an increase in HH was not observed after the HH promotion campaign [31]. Results from this study were shared with administrative and clinical leaders from both study sites. Findings have informed integrated hand hygiene training and monitoring programs and led to greater awareness of the importance of and barriers to adequate hand hygiene practices at both hospitals. One of the facilities plans to continue local production of ABHR following the conclusion of the study. The other has decided to purchase disinfecting gel rather than continue producing ABHR due to cost, supply chain and human resources constraints.

## Conclusion

Our intervention demonstrated that through local production and distribution of ABHR, hospitals in LMICs can increase the availability of functional HH resources at the point of care. However, in our study at two hospitals in DR, increased availability of HHRs did not lead to an increase in HHA among HCWs even when paired with a multimodal HH promotion campaign. Our findings suggest that the most powerful driver of HHA among HCWs may be the real or perceived risk to themselves and their households. Our results suggest that educational efforts should emphasize the importance of HH even when gloves are used. Further research is needed to determine if these findings are generalizable to other settings.

## Supplementary Material

Supporting info - table S1 Appendix. Predictors of hand hygiene adherence among healthcare workers at two hospitals in the Dominican Republic excluding the effect of glove use.

Supporting info - data S1 Data. Minimal dataset.

## Figures and Tables

**Table 1.** Hand hygiene resources (HHR) in patient-care areas (PCA) at baseline and after an ABHR production and distribution intervention.

Hand Hygiene Resource Access	Baseline 4/6/21−9/23/21	Post-intervention 1/11/23−3/16/23	p-value
Number of PCAs evaluated, N			
Total	169	189	NA
Hospital 1	92	85	NA
Hospital 2	77	104	NA
Any HHR, N (%)			
Total	156 (92)	180 (95)	0.200
Hospital 1	85 (92)	79 (93)	0.900
Hospital 2	71 (92)	101 (97)	0.200
Functional HHR, N (%)			
Total	79 (47)	173 (92)	<**0.001**
Hospital 1	62 (67)	77 (91)	<**0.001**
Hospital 2	17 (22)	96 (92)	<**0.001**
Any HWS*, N (%)			
Total	148 (88)	151 (80)	0.051
Hospital 1	77 (84)	65 (76)	0.200
Hospital 2	71 (92)	86 (83)	0.062
Functional HWS, N (%)			
Total	64 (38)	77 (41)	0.600
Hospital 1	51 (55)	39 (46)	0.200
Hospital 2	13 (17)	38 (37)	0.004
Any ABHR dispenser, N (%)			
Total	52 (31)	169 (89)	<**0.001**
Hospital 1	47 (52)	74 (87)	<**0.001**
Hospital 2	5 (6.5)	95 (91)	<**0.001**
Functional ABHR dispenser, N (%)			
Total	43 (25)	167 (88)	<**0.001**
Hospital 1	39 (42)	73 (86)	<**0.001**
Hospital 2	4 (5.2)	94 (90)	<**0.001**

* Hand-Washing Station

**Table 2.** Hand hygiene adherence and glove use during patient contact among healthcare workers, Dominican Republic.

	Before Contact N (%)						After Contact N (%)						Composite N (%)	
	Total	ABHR	HW^1^	Gloves	HHA	Gloves and HHA	Total	ABHR	HW	Gloves^2^	HHA	Gloves and HHA^3^	Total	HHA^4^
Total	2025	109 (5.4)	41 (2.0)	802 (39.6)	146 (7.2)	15 (0.7)	2011	296 (14.7)	328 (16.3)	792 (39.4)	615 (30.6)	285 (14.2)	4036	761 (18.9)
Round 1	680	42 (6.2)	19 (2.8)	304 (44.7)	61 (9.0)	2 (0.3)	674	81 (12.0)	171 (25.4)	299 (44.3)	250 (37.1)	122 (18.1)	1354	311 (23.0)
Round 2	687	22 (3.2)	14 (2.0)	251 (36.5)	33 (4.8)	4 (0.6)	679	96 (14.2)	99 (14.6)	246 (36.2)	187 (27.6)	85 (12.5)	1366	223 (16.3)
Round 3	658	45 (6.8)	8 (1.2)	247 (37.5)	52 (7.9)	9 (1.4)	658	117 (17.8)	53 (8.1)	247 (37.5)	175 (26.6)	78 (11.9)	1316	226 (17.2)

1— Handwashing with water and soap.

2— Since we only recorded the donning of new gloves before patient contact, this column indicates the number and percentage of patient contacts without missing data for after-contact HHA where gloves were donned before patient contact.

3— Since we only recorded the donning of new gloves before patient contact, this column indicates the number and percentage of patient encounters where gloves were donned prior to patient contact, and HH was practiced after patient contact.

4— Percent = N Hand Hygiene Opportunities with Hand Hygiene Adherence divided by Total N Hand Hygiene Opportunities.

**Table 3.** Predictors of hand hygiene adherence among healthcare workers, Dominican Republic.

		Hand Hygiene Adherence, N (%)		Univariate Analysis		Multivariate Analysis^1^	
		No	Yes	OR^2^ (95% CI^3^)	p-value	aOR^4^ (95% CI)	p-value
Round	1	1043 (77.0)	311 (23.0)	-	-	-	-
	2	1143 (83.7)	223 (16.3)	0.65 (0.54−0.79)	<0.001	0.62 (0.38−1.03)	0.066
	3	1089 (82.8)	227 (17.2)	0.70 (0.58−0.85)	<0.001	**0.59 (0.36−0.95)**	**0.029**
Relationship to patient contact	After	1396 (69.4)	615 (30.6)	-	-	-	-
	Before	1879 (92.8)	146 (7.2)	0.18 (0.14−0.21)	<0.001	**0.17 (0.12−0.24)**	<**0.001**
Glove Use	No	1979 (81.2)	459 (18.8)	-	-	-	-
	Yes	1294 (81.2)	300 (18.8)	1.00 (0.85−1.17)	0.996	**0.80 (0.64−1.00)**	**0.049**
Contact Type	Invasive	1200 (80.4)	293 (19.6)	-	-	-	-
	Non-invasive	2057 (81.5)	466 (18.5)	0.93 (0.79−1.09)	0.366	**0.61 (0.46−0.81)**	**0.001**
Profession	Doctor	1220 (77.6)	353 (22.4)	-	-	-	-
	Nurse	2011 (83.3)	404 (16.7)	0.69 (0.59−0.81)	<0.001	0.78 (0.57−1.06)	0.111
	Other	44 (91.7)	4 (8.3)	0.31 (0.09−0.78)	0.028	**0.23 (0.06−0.95)**	**0.042**
Hospital	1	1664 (84.2)	312 (15.8)	-	-	-	-
	2	1611 (78.2)	449 (21.8)	1.49 (1.27−1.75)	<0.001	**1.64 (1.06−2.54)**	**0.027**

1- Variables included: Round, Relationship to patient contact, Glove use, Contact type, Profession, Hospital

2- Odds ratio

3- Confidence interval

4- Adjusted odds ration

**Table 4.** Predictors of new glove use before patient contact among healthcare workers, Dominican Republic.

		Glove Use, N (%)		Univariate Analysis		Multivariate Analysis	
		No	Yes	OR (95% CI)	p	aOR (95% CI)	p
Round	1	376 (55.3)	304 (44.7)	-	-	-	-
	2	436 (63.5)	251 (36.5)	0.71 (0.57−0.88)	0.002	**0.68 (0.48−0.98)**	**0.037**
	3	411 (62.5)	247 (37.5)	0.74 (0.60−0.92)	0.008	0.89 (0.53−1.49)	0.651
Contact Type	Invasive	437 (58.3)	312 (41.7)	-	-	-	-
	Non-invasive	780 (61.6)	486 (38.4)	0.87 (0.73−1.05)	0.147	**0.21 (0.10−0.44)**	<**0.001**
Profession	Doctor	574 (72.8)	215 (27.2)	-	-	-	-
	Nurse	639 (52.7)	573 (47.3)	2.39 (1.98−2.91)	<0.001	**7.12 (3.02−16.79)**	<**0.001**
	Other	10 (41.7)	14 (58.3)	3.74 (1.65−8.79)	0.002	3.02 (0.87−10.50)	0.083
HCF	1	782 (79.2)	205 (20.8)	-	-	-	-
	2	441 (42.5)	597 (57.5)	5.15 (4.25−6.30)	P<0.001	**30.39 (9.89−93.38)**	<**0.001**

## Data Availability

The dataset underlying all quantitative findings from this study will be made available through the PLoS Water website.

## References

[1] World Health Organization. Report on the burden of endemic health care-associated infection world-wide [Internet]. Geneva: World Health Organization; 2011 [cited 2023 Jun 23]. 40 p. Available from: https://apps.who.int/iris/handle/10665/80135

[2] Lotfinejad N, Peters A, Tartari E, Fankhauser-Rodriguez C, Pires D, Pittet D. Hand hygiene in health care: 20 years of ongoing advances and perspectives. Lancet Infect Dis. 2021 Aug 1; 21(8):e209–21. 10.1016/S1473-3099(21)00383-2 PMID: 34331890 34331890

[3] Pessoa-Silva CL, Hugonnet S, Pfister R, Touveneau S, Dharan S, Posfay-Barbe K, Reduction of health care associated infection risk in neonates by successful hand hygiene promotion. Pediatrics. 2007 Aug; 120(2):e382–390. 10.1542/peds.2006-3712 PMID: 17664257 17664257

[4] World Health Organization, Evidence of hand hygiene to reduce transmission and infections by multidrug resistant organisms in health-care settings, 2014.

[5] Pittet D, Hugonnet S, Harbarth S, Mourouga P, Sauvan V, Touveneau S, Effectiveness of a hospital-wide programme to improve compliance with hand hygiene. Infection Control Programme. Lancet Lond Engl. 2000 Oct 14; 356(9238):1307–12. 10.1016/s0140-6736(00)02814-2 PMID: 11073019 11073019

[6] World Health Organization, WHO Patient Safety. WHO guidelines on hand hygiene in health care. 2009; (WHO/IER/PSP/2009/01):262. 23805438

[7] Erasmus V, Daha TJ, Brug H, Richardus JH, Behrendt MD, Vos MC, Systematic review of studies on compliance with hand hygiene guidelines in hospital care. Infect Control Hosp Epidemiol. 2010 Mar; 31(3):283–94. 10.1086/650451 PMID: 20088678 20088678

[8] MoorD, RobbinG, QuinJ, ArbogasW. The impact of COVID-19 pandemic on hand hygiene performance in hospitals. Am J Infect Control. 2021 Jan; 49 (1):30–3. 10.1016/j.ajic.2020.08.021 PMID: 32818577 32818577 PMC7434409

[9] Wang Y, Yang J, Qiao F, Feng B, Hu F, Xi ZA, Compared hand hygiene compliance among healthcare providers before and after the COVID-19 pandemic: A rapid review and meta-analysis. Am J Infect Control. 2022 May; 50(5):563–71. 10.1016/j.ajic.2021.11.030 PMID: 34883162 34883162 PMC8648372

[10] Labarca J, Zambrano A, Niklitschek S, Ferrés M, Pérez C, Rabagliati R, H1N1 pandemic influenza impact on hand hygiene and specific precautions compliance among healthcare workers. J Hosp Infect. 2011 Oct; 79(2):177–9. 10.1016/j.jhin.2011.06.003 PMID: 21816507 21816507

[11] dos Santos RP, Konkewicz LR, Nagel F, Lisboa T, Jacoby T, Gastal SL, l. The 2009 H1N1 Influenza A Pandemic and Hand Hygiene Practices in a Hospital in the South of Brazil. Infect Control Hosp Epidemiol. 2010 Dec; 31 (12):1313–5. 10.1086/657582 PMID: 21047186 21047186

[12] Allegranzi B, Bagheri Nejad S, Combescure C, Graafmans W, Attar H, Donaldson L, Burden of endemic health-care-associated infection in developing countries: systematic review and meta-analysis. Lancet Lond Engl. 2011 Jan 15; 377(9761):228–41. 10.1016/S0140-6736(10)61458-4 PMID: 21146207 21146207

[13] Cronk R, Bartram J. Environmental conditions in health care facilities in low- and middle-income countries: Coverage and inequalities. Int J Hyg Environ Health. 2018 Apr; 221(3):409–22. 10.1016/j.ijheh.2018.01.004 PMID: 29352706 29352706

[14] Water and Sanitation | Department of Economic and Social Affairs [Internet]. [cited 2023 Sep 25]. Available from: https://sdgs.un.org/topics/water-and-sanitation

[15] Tusabe F, Nanyondo J, Lozier MJ, Kesande M, Tumuhairwe O, Watsisi M, Improving Access to WHO Formulations of Alcohol-Based Hand Rub in Healthcare Facilities: A District-Wide Approach. Am J Trop Med Hyg. 2023 Jul; 109(1):191–200. 10.4269/ajtmh.22-0554 PMID: 37188343 37188343 PMC10324005

[16] World Health Organization. Guide to Local Production: WHO-recommended Handrub Formulations. 2010.

[17] Padilla P, Ly P, Dillard R, Boukovalas S, Zapata-Sirvent R, Phillips LG. Medical Tourism and Postoperative Infections: A Systematic Literature Review of Causative Organisms and Empiric Treatment. Plast Reconstr Surg. 2018 Dec; 142(6):1644–51. 10.1097/PRS.0000000000005014 PMID: 30489537 30489537

[18] Johns Hopkins University of Medicine, Coronavirus Resource Center, https://coronavirus.jhu.edu/region/dominican-republic, accessed 1 July 2023.

[19] Craig C, Schnorr CD, Then Paulino CJ, Payano EC, Martínez Guzmán P, Ripkey C, Hand hygiene perceptions, preferences, and practices among hospital staff in Dominican Republic in the context of COVID-19: A Qualitative Study. Infection Prevention in Practice. preprint. 2024; 10.1016/j.infpip.2024.100367 PMC11101936 38765916

[20] Berendes D, Martinsen A, Lozier M, Rajasingham A, Medley A, Osborne T, Improving water, sanitation, and hygiene (WASH), with a focus on hand hygiene, globally for community mitigation of COVID-19. PLOS Water. 2022 Jun 15; 1(6):e0000027. 10.1371/journal.pwat.0000027 PMID: 38410139 PMC10896259 38410139

[21] Vega Ocasio D, Berendes D, Salah Z, Garzaro P, Monroe Ramay B, Fahsen N, Characteristics of hand hygiene adherence in healthcare settings in Central America and East Africa—2020–2021. pre-print. 2023;

[22] Chang CN, Reisinger HS, Schweizer ML, Jones I, Chrischilles E, Chorazy M, . Hand Hygiene Compliance at Critical Points of Care. Clin Infect Dis Off Publ Infect Dis Soc Am. 2021 Mar 1; 72 (5):814–20. 10.1093/cid/ciaa130 PMID: 32034404 32034404

[23] Erasmus V, Brouwer W, van Beeck EF, Oenema A, Daha TJ, Richardus JH, A qualitative exploration of reasons for poor hand hygiene among hospital workers: lack of positive role models and of convincing evidence that hand hygiene prevents cross-infection. Infect Control Hosp Epidemiol. 2009 May; 30(5):415–9. 10.1086/596773 PMID: 19344264 19344264

[24] Korniewicz DM, El-Masri M. Exploring the factors associated with hand hygiene compliance of nurses during routine clinical practice. Appl Nurs Res ANR. 2010 May; 23(2):86–90. 10.1016/j.apnr.2008.06.002 PMID: 20420995 20420995

[25] Souza D de O. Health of nursing professionals: workload during the COVID-19 pandemic. Rev Bras Med Trab Publicacao Of Assoc Nac Med Trab-ANAMT. 2021 Mar 3; 18(4):464–71. 10.47626/1679-4435-2020-600 PMID: 33688329 PMC7934175 33688329

[26] Khatib M, Jamaleddine G, Abdallah A, Ibrahim Y. Hand washing and use of gloves while managing patients receiving mechanical ventilation in the ICU. Chest. 1999 Jul; 116(1):172–5. 10.1378/chest.116.1.172 PMID: 10424522 10424522

[27] Thompson BL, Dwyer DM, Ussery XT, Denman S, Vacek P, Schwartz B. Handwashing and glove use in a long-term-care facility. Infect Control Hosp Epidemiol. 1997 Feb; 18(2):97–103. 10.1086/647562 PMID: 9120250 9120250

[28] Eveillard M, Joly-Guillou ML, Brunel P. Correlation between glove use practices and compliance with hand hygiene in a multicenter study with elderly patients. Am J Infect Control. 2012 May; 40(4):387–8. 10.1016/j.ajic.2011.05.008 PMID: 21864940 21864940

[29] Gon G, de Bruin M, de Barra M, Ali SM, Campbell OM, Graham WJ, . Hands washing, glove use, and avoiding recontamination before aseptic procedures at birth: A multicenter time-and-motion study conducted in Zanzibar. Am J Infect Control. 2019 Feb; 47(2):149–56. 10.1016/j.ajic.2018.07.021 PMID: 30293743 30293743 PMC6367567

[30] Smiddy MPRO, Creedon SA. Systematic qualitative literature review of health care workers’ compliance with hand hygiene guidelines. Am J Infect Control. 2015 Mar 1; 43(3):269–74. 10.1016/j.ajic.2014.11.007 PMID: 25728153 25728153

[31] Baseman JG, Revere D, Painter I, Toyoji M, Thiede H, Duchin J. Public health communications and alert fatigue. BMC Health Serv Res. 2013 Aug 5; 13(1):295. 10.1186/1472-6963-13-295 PMID: 23915324 23915324 PMC3751004

