# Supplementary material for: Longitudinal changes in hand hygiene adherence among healthcare workers during the COVID-19 pandemic, Dominican Republic
Source: PLOS Water. Author manuscript; Available in PMC 2025 May 12. (PMC12067931; doi:10.1371/journal.pwat.0000231)
Supplement: Supporting info - table — S1 Appendix. Predictors of hand hygiene adherence among healthcare workers at two hospitals in the Dominican Republic excluding the effect of glove use. [file NIHMS2059951-supplement-Supporting_info_-_table.docx]

**Appendix: Predictors of Hand Hygiene Adherence Among Healthcare Workers at Two Hospitals in the Dominican Republic Excluding the Effect of Glove Use**

|  | **Independent variable** | **Total (N)** | **Used ABHS**^[[1]](#footnote-1)^ **or HW**^[[2]](#footnote-2)^**, N(%)** | | **Univariate Analysis** | | **Multivariate Analysis** | |
| --- | --- | --- | --- | --- | --- | --- | --- | --- |
|  |  |  | **No** | **Yes** | **OR**^[[3]](#footnote-3)^ **(95% CI**^[[4]](#footnote-4)^**)** | **p** | **aOR**^[[5]](#footnote-5)^ **(95% CI)** | **p** |
| **Round** | 1 | 1354 | 1043 (77.0) | 311 (23.0) | - | - | - | **-** |
|  | 2 | 1366 | 1143 (83.7) | 223 (16.3) | 0.65 (0.54-0.79) | **<0.001** | 0.64 (0.39-1.05) | 0.078 |
|  | 3 | 1316 | 1089 (82.8) | 227 (17.2) | 0.70 (0.58-0.85) | **<0.001** | 0.60 (0.38-0.96) | **0.031** |
| **Relationship to patient contact** | After contact | 2011 | 1396 (69.4) | 615 (30.6) | - | - | - | - |
|  | Before contact | 2025 | 1879 (92.8) | 146 (7.2) | 0.18 (0.14-0.21) | **<0.001** | 0.17 (0.12-0.24) | **<0.001** |
| **Contact type** | Invasive | 1493 | 1200 (80.4) | 293 (19.6) | - | - | - | - |
|  | Non-invasive | 2523 | 2057 (81.5) | 466 (18.5) | 0.93 (0.79-1.09) | 0.366 | 0.65 (0.48-0.87) | **0.004** |
| **Profession** | Doctor | 1573 | 1220 (77.6) | 353 (22.4) | - | - | - | - |
|  | Nurse | 2415 | 2011 (83.3) | 404 (16.7) | 0.69 (0.59-0.81) | **<0.001** | 0.74 (0.55-0.98) | **0.036** |
|  | Other | 48 | 44 (91.7) | 4 (8.3) | 0.31 (0.09-0.78) | **0.028** | 0.22 (0.056-0.88) | **0.032** |
| **Hospital** | 1 | 1976 | 1664 (84.2) | 312 (15.8) | - | - | - | - |
|  | 2 | 2060 | 1611 (78.2) | 449 (21.8) | 1.49 (1.27-1.75) | **0.001** | 1.64 (1.06-2.54) | **0.027** |

1. Alcohol-based hand rub [↑](#footnote-ref-1)
2. Hand-washing with soap [↑](#footnote-ref-2)
3. Odds-ratio [↑](#footnote-ref-3)
4. Confidence -interval [↑](#footnote-ref-4)
5. Adjusted odds ratio [↑](#footnote-ref-5)
